# Computer simulation of partitioning of ten pentapeptides Ace-WLXLL at the cyclohexane/water and phospholipid/water interfaces

**DOI:** 10.1186/1471-2091-6-30

**Published:** 2005-12-20

**Authors:** Marcela P Aliste, D Peter Tieleman

**Affiliations:** 1 Department of Biological Sciences, University of Calgary, 2500 University Dr. NW, Calgary, Alberta T2N 1N4, Canada; 2 School of Biology, Georgia Institute of Technology, 310 Ferst Dr NW, Atlanta, Georgia, 30332, USA

## Abstract

**Background:**

Peptide-membrane interactions play a key role in the binding, partitioning and folding of membrane proteins, the activity of antimicrobial and fusion peptides, and a number of other processes. To gain a better understanding of the thermodynamics of such interactions, White and Wimley created an interfacial hydrophobicity scale based of the transfer free energy from water to octanol or lipid bilayers of a series of synthetic peptapeptides (Ace-WLXLL, with X being any of the twenty natural amino acids) (White and Wimley (1996) *Nat. Struct. Biol. 3*, 842–848). In this study, we performed molecular dynamics simulations of a representative set of ten of these peptides (X = D, K, R, N, A, T, S, I, F and W) in two membrane mimetic interfaces: water-cyclohexane (10 ns) and a fully solvated dioleoylphosphatidylcholine (DOPC) bilayer (50 ns) using both constant pressure and constant area ensembles. We focus on partitioning of the ten peptides at the cyclohexane/water and lipid/water interfaces.

**Results:**

The peptides rapidly equilibrate (< 2 ns) and partition at the cyclohexane/water interface. The X3 guest residue assumes average orientations that depend on the nature of the side chain. At the DOPC/water interface, dynamics is much slower and convergence is difficult to achieve on a 50 ns timescale. Nonetheless, all peptides partition to the lipid/water interface with distributions with widths of 1–2 nm. The peptides assume a broad range of side chain and backbone orientations and have only a small effect on the area of the unit cell. On average, hydrophobic guest residues partition deeper into the hydrophobic core than hydrophilic residues. In some cases the peptides penetrate sufficiently deep to somewhat affect the distribution of the C=C double bond in DOPC. The relative distribution of the X3 guest residue compared to W1 and L5 is similar in the water/cyclohexane and water/lipid simulations. Snapshots show mostly extended backbone conformations in both environments. There is little difference between simulations at a constant area of 0.66 nm^2 ^and simulations at constant pressure that approximately yield the same average area of 0.66 nm^2^.

**Conclusion:**

These peptides were designed to assume extended conformations, which is confirmed by the simulations. The distribution of the X3 side chain depends on its nature, and can be determined from molecular dynamics simulations. The time scale of peptide motion at a phospholipids-water interface is too long to directly calculate the experimentally measured hydrophobicity scale to test and improve the simulation parameters. This should be possible at the water/cyclohexane interface and likely will become feasible in the future for the phospholipids/water case.

## Background

The interactions of membrane-active peptides with lipids are of basic interest in a range of biological processes [1], including membrane fusion [2], the action of antimicrobial peptides [3], and lipid recognition by membrane binding domains in larger proteins [4]. A precise thermodynamic description of such interactions is crucial for understanding membrane protein folding. Systematic series of model peptides are an excellent tool to gain insight in the effect of different side chains on partitioning of peptides and membrane proteins. Wimley and White have created a hydrophobicity scale for interfacial partitioning based on the pentapeptides Ace-WLXLL, where X stands for all 20 naturally occurring amino acids [5].

In a previous paper, we have investigated the properties of Ace-WLRLL and Ace-WLKLL, with an emphasis on salt-bridge formation between the charged Arg or Lys side chain with the C-terminus [6]. In this paper we extend these simulations to 10 different peptides, with different side chain properties for residue 3: hydrophilic, hydrophobic, anionic, cationic, or aromatic. We study the behavior of this set of peptides at the water/cyclohexane and the water/phospholipid interface.

Our primary questions are: where do the peptides partition at the water/hydrophobic interface, and can we distinguish statistically significant differences between the different peptides?

The location and structure of the peptides is relevant for a molecular interpretation of the thermodynamic hydrophobicity scale. These well-characterized peptides are also useful models for a broad range of antimicrobial peptides that are thought to interact at the lipid/water interface [3]. Finally, computer simulations are becoming an extremely popular tool to study membrane proteins and interactions between lipids and membrane proteins [7-9], but the amount of accurate experimental data that can be used to critically test simulations of lipid-protein interactions is very limited. It is important to understand the strengths and limitations of computer simulations to study such peptides. The present set of simulations addresses the question of timescales involved in the equilibration and dynamics of peptides at different interfaces. It also shows that there are no significant differences between simulations of the DOPC bilayer with constant area and constant pressure, probably because the chosen constant area is approximately the same as the area obtained from the constant pressure simulations.

A long-term goal of the work presented in this study is to be able to calculate the relative free energies of transfer for different side chains in such a way that the calculations are directly comparable to the experiments. Although we show here that this is currently not feasible using standard free-energy methods (e.g. [10]) for the case of the lipid/water interface, it is likely this will become feasible with improved simulation algorithms and faster computers in the coming years. In addition, simulations on these well-characterized peptides may help to establish protocols to study less characterized but biologically more important peptides.

## Results

**Figure 1:**
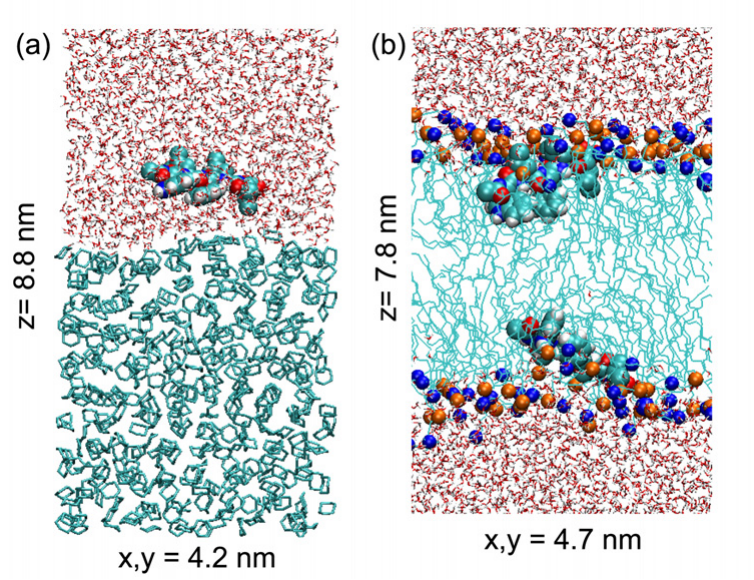
Overview of the simulation systems. Starting configurations: (a) the water/cyclohexane system and (b) the DOPC bilayer. Phosphorous atoms are shown in orange, nitrogen atoms in blue. The peptides are shown in a space-filling representation.

Figure 1 shows the general system setup of both the cyclohexane and lipid simulations. We describe first the orientation and distribution of the pentapeptides at the water/cyclohexane interface. Then we analyze the orientation and distribution at the lipid/water interface as well as the orientation of the Trp residue. In a previous study we carried out very detailed analyses of the peptide backbone, including backbone dihedrals and cluster analyses, on the two peptides that are able to form intramolecular salt bridges [6]. Almost all structural variation in these peptides was due to the presence or absence of a salt bridge. We do not describe the peptide structure in detail here but note that the backbone of these peptides is mostly extended in all simulations.

### Orientation of the peptides at the cyclohexane/water interface

**Figure 2:**
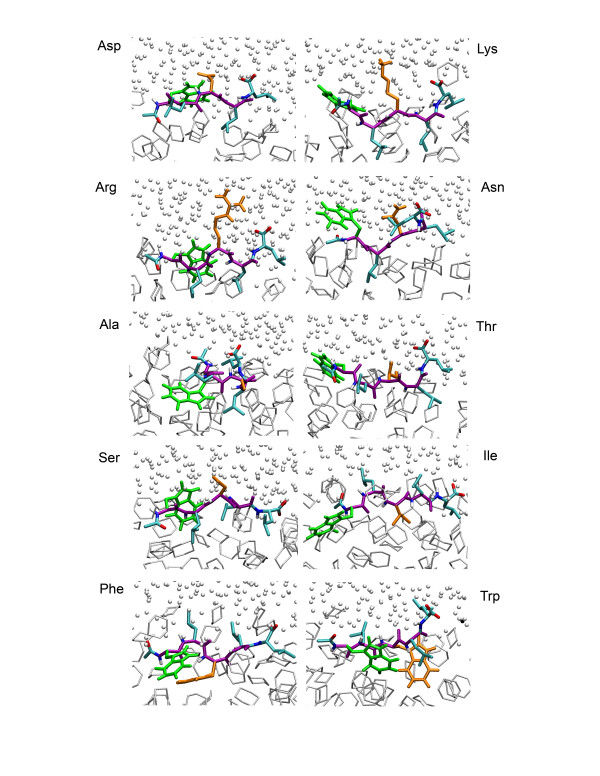
Snapshots of the ten peptides in the water/cyclohexane system after 10 ns. The peptide backbone is violet, the Ace and C-terminus are colored by atom type, Trp is green, Leu cyan, and the changing residue X3 is orange, while the water is shown as small white spheres and the cyclohexane is shown in light grey.

**Figure 3:**
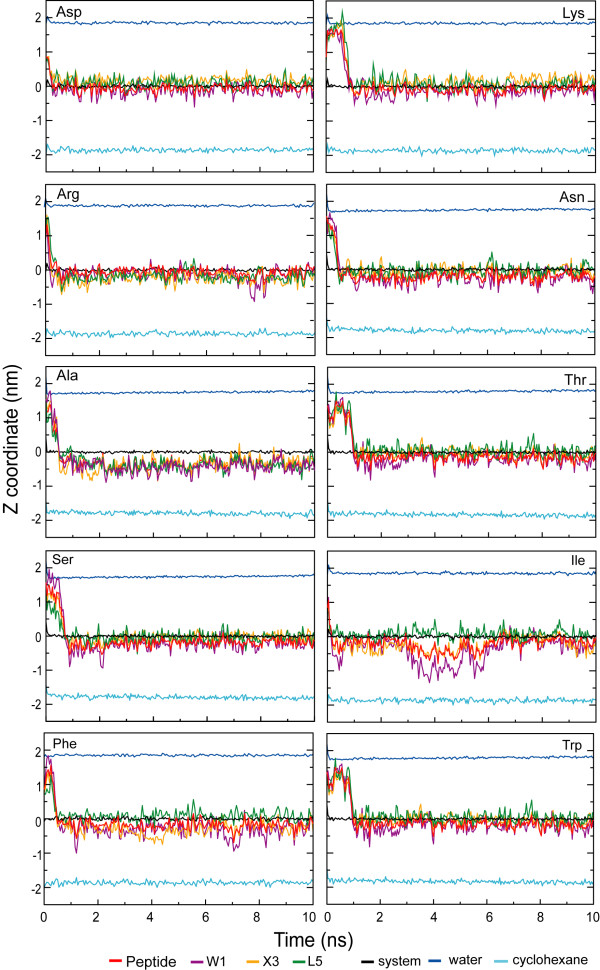
The z-coordinates of the center of mass of the entire peptide, three side chains, water and cyclohexane as function of time for all ten cyclohexane/water systems. Trajectory profiles along the z-coordinates for the peptides in the cyclohexane/water systems. The zeropoint is the point where the water and cyclohexane densities are equal (see figure 4).

At the cyclohexane/water interface the peptides rapidly find a preferred depth, after which they fluctuate around this position. Figure 2 shows snapshots of each of the ten peptides after ten nanoseconds of simulation. Although these are single snapshots, it is clear that all peptides have a well-defined orientation, with leucine side chains in the cyclohexane phase, tryptophan side chains predominantly in the cyclohexane phase, and hydrophilic side chains in water. The backbone of the peptides is predominantly extended. Movie 1 (see Additional file 1) shows in detail the dynamics of one of the ten peptides. Figure 3 shows the center of mass of each of the peptides as function of time, as well as the centers of mass of three of the side chains, the cyclohexane, and water. The initial movement of the peptides from their starting location in the water phase to the water/cyclohexane interface occurs mostly in the first nanosecond, followed by a stable location of the peptide as whole. The side chains show somewhat larger fluctuations around their average depth compared to the entire peptide, but overall the peptides are firmly anchored at the interface.

### Distribution of the side chains at the cyclohexane/water interface

**Figure 4:**
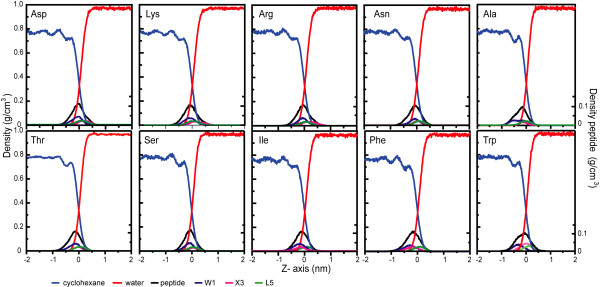
Density profiles along the z-axis. The density profiles of the peptides have been averaged over the last 2 ns of simulation. The scales on the left and right axes correspond to the solvent components (water and cyclohexane) and peptides and/or side chains, respectively. The middle of the interface (0 nm) is defined as the point where the partial density of the water equals the partial density of the cyclohexane.

Figure 4 shows the density profiles for each of the ten peptides, a measure of the distribution of the peptides and their side chains at the interface. The water/cyclohexane interface has a width of only a few tens of a nanometer and is essentially molecularly sharp [11]. Interestingly, the peptide sits right at the interface and significantly perturbs the cyclohexane distribution, which is smooth without a peptide (not shown). W1, the first tryptophan, is most deeply embedded in the cyclohexane, while L5 with its terminal carboxyl group is stretched across the interface. The most hydrophobic X3 residues, Ile and Phe, are on average deeper inside the cyclohexane than L5, while the most hydrophilic residues (Arg, Asn) are deeper into the water phase.

### Orientation of the peptides at the DOPC/water interface

**Figure 5:**
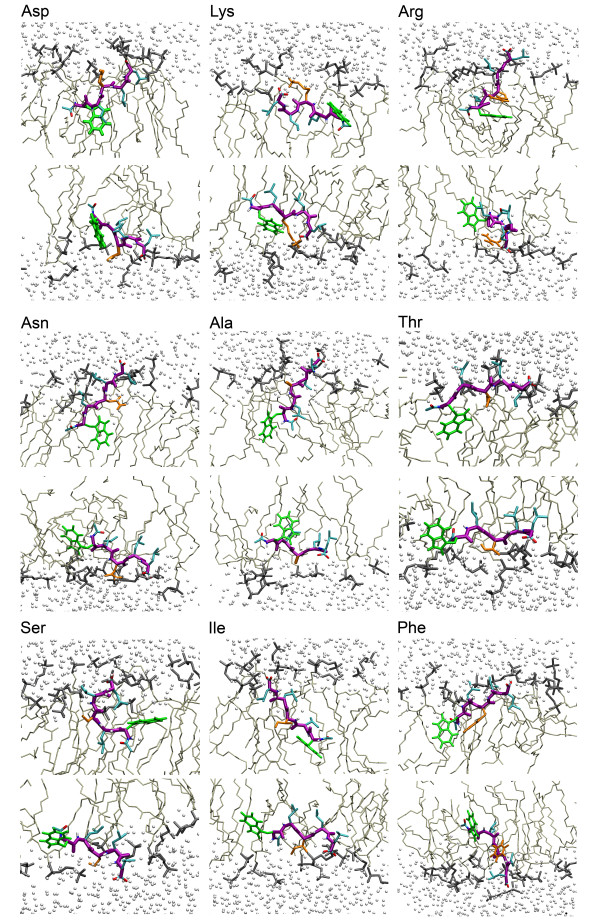
A. Snapshots of all peptides except the Trp peptide in the DOPC lipid bilayer system at 50 ns (NpT simulations). The first row and the third represent the peptide in the upper leaflet; the second and the last row the peptide in the lower leaflet. The peptide backbone is violet, the Ace and C-terminus are colored by atom type, Trp is green, Leu cyan, and the changing residue X3 is orange, while the water is shown as small white spheres and the cyclohexane is shown in light grey. The water molecules are shown as white spheres. The DOPC lipid bilayer is shown as two separated groups: the head group (choline and phosphate) is dark grey while the tails are lighter gray.

**Figure 6:**
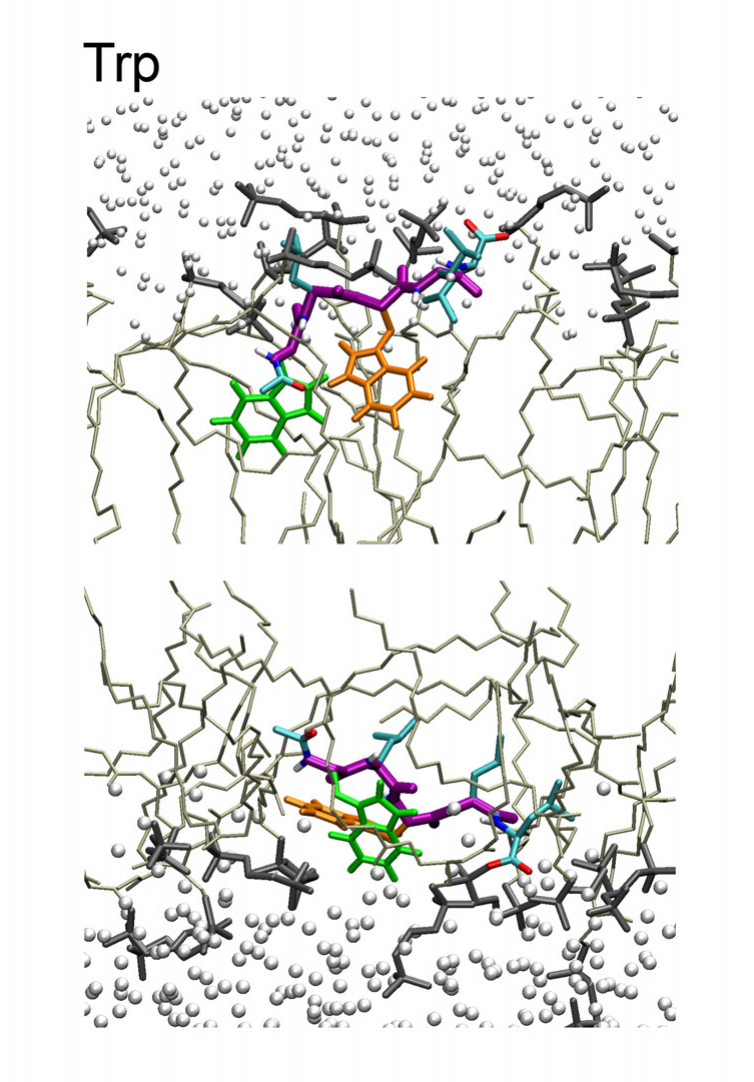
A close-up of the Trp peptide, in the same representation as the other 9 peptides in Figure 5.

The picture for the DOPC/water interface is more complicated. The interface itself is quite broad, comparable to the width of the distribution of the peptide at the sharp cyclohexane/water interface. White and co-workers have previously pointed out that the interfacial width of ca. 1.5 nm is wide enough to accommodate a standard alpha-helix [12]. Figures 5 and 6 show snapshots of each of the peptides after 50 ns of simulation.

**Figure 7:**
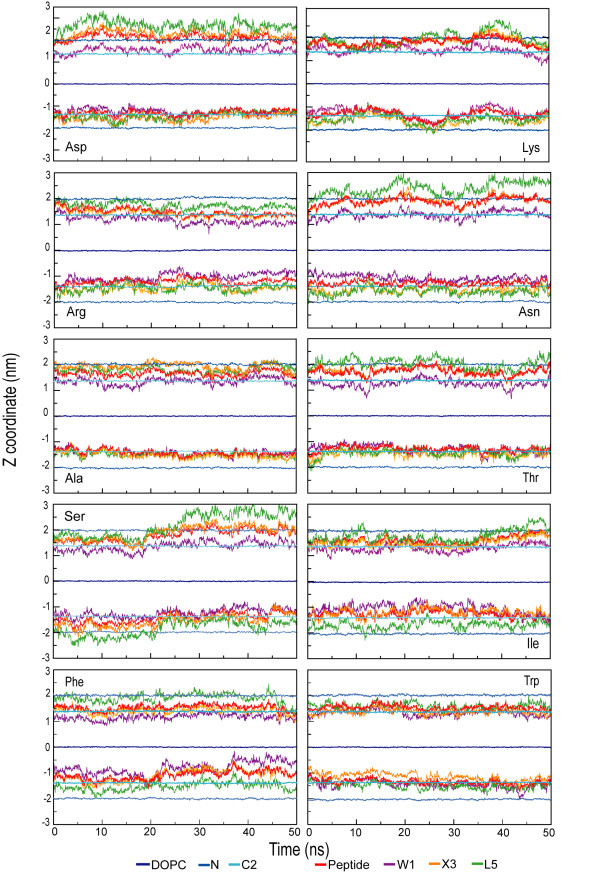
Trajectory profiles along the z-coordinates for the peptides in the lipid bilayer system (NpAT simulations). The z-coordinates for the center of mass of the different groups are plotted averaged over 10 ps. The groups are the two peptides, the three side chains in positions Trp1, X3 and Leu5 with respect to the average center of mass (0 nm) of the DOPC group. The lipid bilayer interface is defined with the coordinates: the choline group (N) and the first CH_2 _group of the lipid chain (the second carbon counting from the ester link, C2).

**Figure 8:**
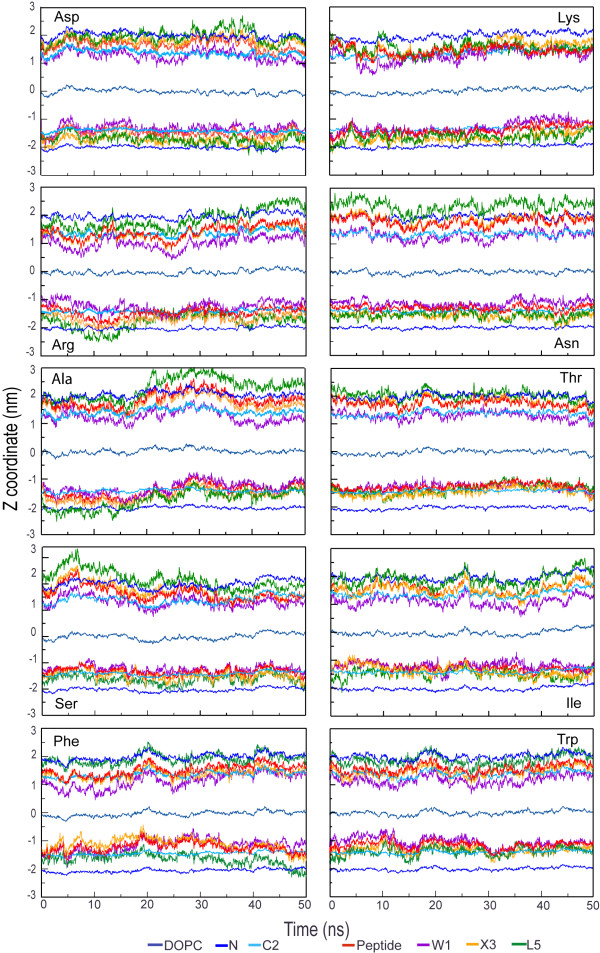
Trajectory profiles along the z-coordinates for the peptides in the lipid bilayer system (NpT simulations). The z coordinates for the center of mass of the different groups: peptide, three side chains in positions Trp1, X3 and Leu5 with respect to the average center of mass (0 nm) of the DOPC group. The lipid bilayer interface is defined with the coordinates of the choline group (N) and the first CH_2 _group of the lipid chain (the second carbon counting from the ester link, C2).

The snapshots broadly suggest an amphipathic orientation comparable to the orientation in the cyclohexane/water case, although the picture is somewhat obscured by the ability of water to penetrate more deeply into the membrane. The snapshots also necessarily show a somewhat exaggerated view because they are side projections that do not fully do justice to local transient changes in the lipid structure. Movie 2 (see Additional file 2) shows the dynamics of one of the peptides at the lipid/water interface. Figures 7 and 8 show the location along the bilayer normal of the peptides and the 1^st^, 3^rd^, and 5^th ^side chain as a function of time. There appears to be little difference between simulations with constant pressure versus simulations with constant area. In the constant pressure simulations, all peptides remain at the interface as expected and fluctuate slowly in depth, both because of the slow fluctuation of the interface itself and their intrinsic internal and external motion at the interface.

### Distribution of the side chains at the DOPC/water interface

**Figure 9:**
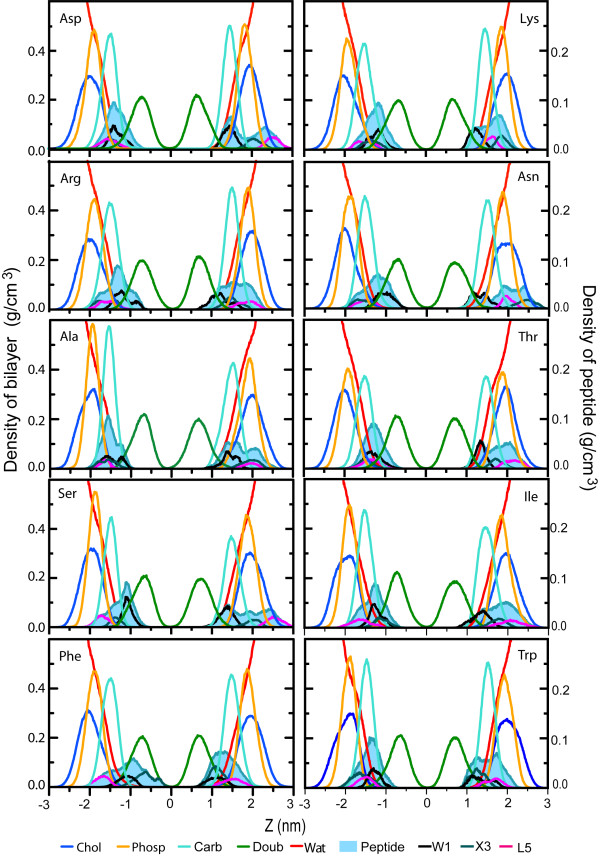
Density profiles along the bilayer normal (NpAT simulations). The profiles were obtained over the last 10 ns of the simulations; the left and right scales of the graph correspond to the lipid components and the peptides and/or side chain, respectively. Partial density for some of the interface components of the lipid bilayers (as choline, phosphate and carbonyl) and one component of the hydrocarbon core of the bilayers, the double bond distribution. Partial densities of the peptides are show as solid representation and the three side chains are shown Trp1, X3 and Leu5.

**Figure 10:**
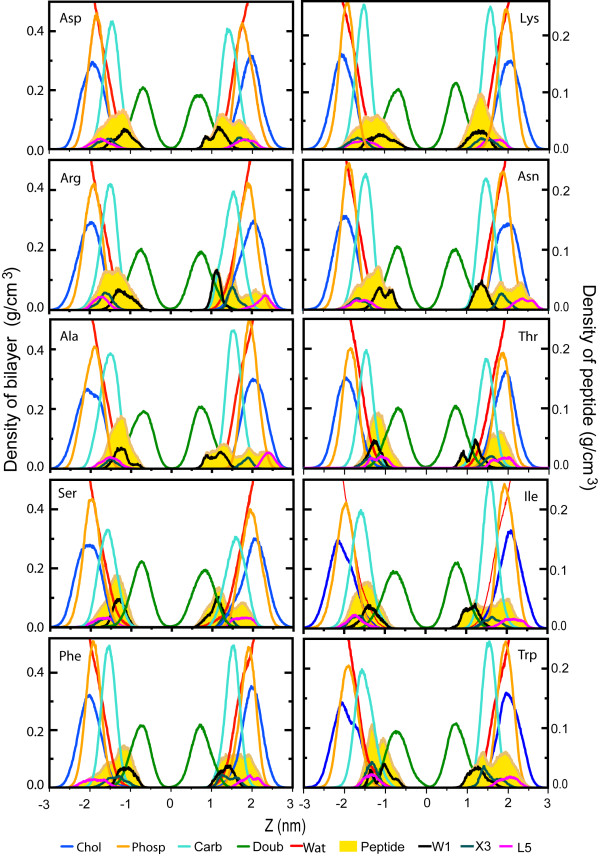
Density profiles along the bilayer normal (NpT simulations). The profiles were obtained over the last 10 ns of the simulations; the left and right scales of the graph correspond to the lipid components and the peptides and/or side chain, respectively. Partial density for some of the interface components of the lipid bilayers (as choline, phosphate and carbonyl) and one component of the hydrocarbon core of the bilayers, the double bond distribution. Partial densities of the peptides are show as solid representation and the three side chains are shown Trp1, X3 and Leu5.

Figures 9 and 10 show the density profiles for the ten peptide simulations at constant area (figure 9) and at constant pressure (figure 10). The density distribution for the upper and lower peptide has not yet converged to the same distribution in most cases, even in 50 ns. This puts a lower limit on lipid-peptide simulations even for small peptides of 50 ns, for clearly amphiphatic peptides that do not get locked in spurious secondary structure. The difference in distributions between the two sets of simulations (NpT and NpAT) does not appear to be significant compared to the difference in distribution between the two peptides within a single simulation.

As in the water/cyclohexane simulations, the W1 residue is located most deeply inside the membrane interior, with L5 usually the furthest inside the water phase. Of particular interest is the location of X3. For charged amino acids, the distribution overlaps with the distribution of L5 and in a few cases even extends further into the water, as in one of the Lys and one of the Asn peptides. Hydrophobic residues are typically located further inside the membrane, with small hydrophilic residues taking intermediate positions.

### Orientation of the Trp ring at the DOPC/water interface

**Figure 11:**
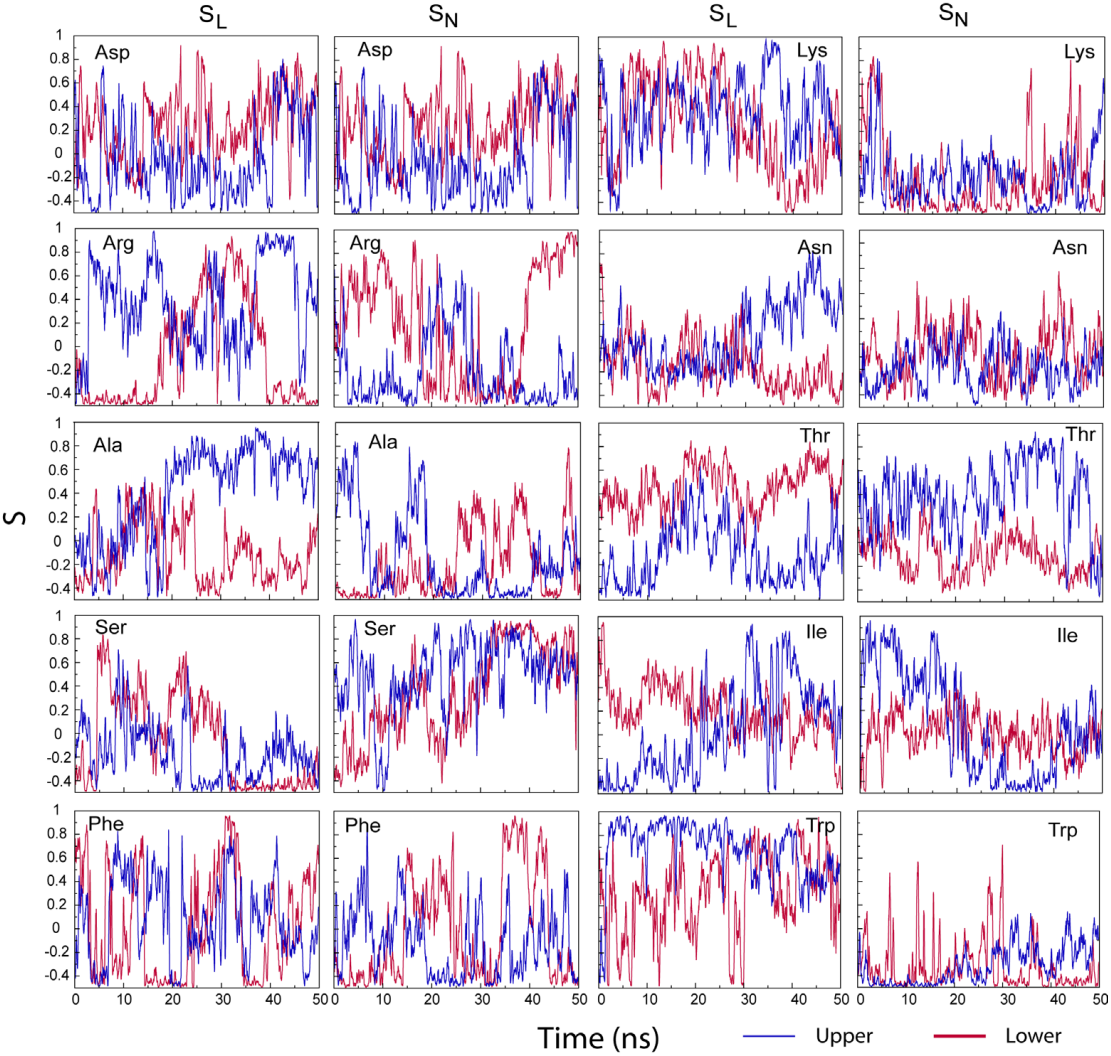
Orientations of the Trp1 ring (NpT simulations) as a function of time, defined by S_L _and S_N_.

Figure 11 shows the orientation of the Trp rings as a function of time in terms of two order parameters [13]. S_L _is an order parameter for the 'long' axis of the Trp side chain and varies between 1 and -0.5. A value of 1 means the long axis of the Trp side chain is aligned with the normal of the membrane, a value of -0.5 means the long axis of the Trp side chain is perpendicular to the normal of the membrane. S_N _is a similar order parameter for the normal on the Trp side chain. Because of the definition of S_L _and S_N _as a square of the cosine of the angle between the L or N molecular axis and the normal on the bilayer the upper and lower peptide should be comparable. It is clear from Figure 11 that the Trp orientations fluctuate quite slowly on a 50 ns time scale and many orientations occur. The distribution also differs per peptide, suggesting that the nature of the X3 residue may affect the orientation of the W1 side chain.

### Constant area versus constant pressure

The constant pressure simulations allow us to investigate the effect of the peptides on the area of the unit cell. Table 1 gives the average area per lipid, calculated from the size of the simulation box L_x _× L_y_/32, where 32 is the number of lipids in each of the two leaflets. Fluctuations in area are significant and the statistical uncertainty in averages calculated over 25 ns is substantial in this small system. The expected differences due to the difference in one of 5 side chains with a peptide that is otherwise the same are small, but it is conceivable that with better averaging we would observe a difference between e.g. Trp and Arg in the 3 position. Table 1 also gives the average lateral pressure for all 10 constant area simulations. In all ten, the average normal pressure is between -3 and +3 bar, with an estimated standard error of 3 bar. In all cases the standard deviation for both the normal and the lateral pressure is hundreds of bar, normal fluctuations caused by the small system size. Peptides with the more hydrophobic residues cause a larger negative pressure, indicating a tendency to contract the box. This agrees with the trend in the areas calculated from the constant pressure simulations. We have not calculated in detail the effect of the peptides on the lipids, but because the areas per lipid show very minor changes we expect only small local perturbations to the lipids that are directly interacting with the hydrophobic side chains that protrude into the membrane interior. Perturbations by small molecules like pentachlorophenol [14] and pyrene [15] were minimal, while major effects of antimicrobial peptides on lipid structure imply major changes in the lipid area [16,17].

**Table 1:** Area per lipid (nm^2^) for the simulations using a NPT ensemble average over last 25 ns of the simulation time and the lateral pressure for the simulations using a NPAT ensemble average over the last 25 ns (area of 0.66 nm^2^).

Peptides	Area (nm^2^)	Average lateral pressure (bar)
Asp	0.682 ± 0.012	-22 ± 3
Lys	0.676 ± 0.009	-18 ± 3
Arg	0.671 ± 0.015	-33 ± 3
Asn	0.676 ± 0.010	-23 ± 3
Ala	0.657 ± 0.020	-45 ± 3
Ser	0.663 ± 0.018	-22 ± 3
Thr	0.666 ± 0.012	-33 ± 3
Ile	0.673 ± 0.020	-53 ± 3
Phe	0.663 ± 0.011	-28 ± 3
Trp	0.668 ± 0.012	-49 ± 3

## Discussion

Overall, the difference in orientation between the different peptides is small, supporting a key assumption of the experimental design of these peptides [5]. At the water/cyclohexane interface sampling is so fast that we can obtain converged distributions quite rapidly. The peptides are clearly able to adopt minor structural changes to allow X3 an orientation consistent with its chemical nature. Although this overall trend is visible in the distributions of the side chains at the water/phospholipids interface, the intrinsic motions of both lipids and the peptide at this interface are too slow to obtain accurate convergence on a 50 ns time scale of hydrophobicity.

Ideally, we would like to make a direct link with the experimentally measured hydrophobicity scale. Although the density profiles can in principle be converted to free energy profiles for the distribution of side chains, in practice this requires computational sampling of orientations that have a low probability. Because such states are not accurately sampled in an equilibrium simulation, the results would be unreliable. It should be possible at the current state of the art in molecular dynamics simulations to calculate a hydrophobicity scale using the water/cyclohexane interface. This is analogous to the use of calculations of free energy of hydration and free energy of transfer between water and cyclohexane for side-chain analogues in recent studies on several commonly used force fields [10,18,19]. A reasonable approach would be to alchemically mutate each X3 side chain to a 'dummy' side chain that does not interact with its environment.

We initially suspected that constant area simulations would make it harder for the peptides to equilibrate at the interface, as this might require fluctuations in the area of the interface. We obtained some anecdotal evidence in simulations where a peptide left the interface and would not reinsert on a reasonable time scale with constant area, but would reinsert with constant pressure. In the final set of simulations, however, the difference between constant area and constant pressure appears minimal. One reason for this is likely that the average area of the interface in both sets of simulations is the same. From a technical point of view, it would be worth examining this matter in more detail at different fixed areas. It is also likely that there would be a size-effect, as a constant small area might be more restrictive than a constant large area for insertion of the same peptide.

The procedures and force field used in this study are in common use and have not failed any critical tests against experiment. We would also expect some possible errors to cancel in a direct comparison between the different peptides within the same simulation setup. One possible source of concern are the errors in the free energy of transfer between water and lipid for the peptide side chains, termini, and backbone. Villa and Mark calculated the free energy of transfer for the side chains of this force field between water and cyclohexane [18]. There is room for improvement of the force field, but the relative order in free energy of transfer for the residues used here is correct, and we expect reasonable distributions of the side chains at the interface. These inaccuracies in the free energy of transfer will be a serious problem in attempts to quantitatively reproduce the experimental hydrophobicity scale, and will have to be addressed. Although we cannot obtain accurate numerical agreement with the experimental values for the hydrophobicity scale from these simulations, we believe the atomistic picture emerging from the present simulations is likely to be accurate and could be considered the best 'structure' of the pentapeptides in their environment available.

## Conclusion

The simulations provide a detailed atomistic picture of the behaviour of these peptides and give insight in the molecular basis of the free energy scale. The peptides are generally extended and have the flexibility to allow the guest residue X3 to interact with either the lipid/cyclohexane or the water phase. Although at the current state of the art in molecular dynamics simulations it is not yet possible to calculate the exact values of this hydrophobicity scale, our results suggest that such calculations become a viable way for force field testing and development in the near future. In future work, we are calculating the hydrophobicity scale for the water/cyclohexane and water/octanol case. While this is a major undertaking with present computers, we expect this will be useful in further refinement of computational models for lipid-peptide interactions.

## Methods

### Simulation setup

Figure 1 shows a representative snapshot of the water/cyclohexane cell and the DOPC bilayer. In the cyclohexane simulation, a single peptide is located at the water/cyclohexane interface. The area of the interface is fixed, and the thickness of the cyclohexane and water phases can adjust. In the DOPC/water simulation, we use one peptide at each of the two interfaces. Because the DOPC is not able to change its thickness in the same way as an isotropic liquid, this ensures there is no artificial asymmetry induced by having a peptide in only one interface. The same setup has been used in several other simulations of peptides interacting with membranes, e.g. [16,17]. The ten pentapeptides simulated are Ace-WLXLL, with X = D, K, R, N, A, T, S, I, F and W, including positively and negatively charged side chains, hydrophobic and hydrophilic side chains, and aromatic side chains. In all cases, the C-terminus has a negative charge. The Arg and Lys side chains have a charge of +1, the Asp side chain has a charge of -1. Because the peptide is expected to partition in the interface, we assume that all ionisable residues are in their default charged states. The results for all peptides, with or without charged residues, show that this is a reasonable assumption. All peptides were initially built in an extended conformation.

#### Peptide in water/cyclohexane biphasic cell

In each simulation, one peptide was placed ca. 1.5 nm away from the center of the water/cyclohexane interface, in the water phase of a pre-equilibrated (10 ns) water/cyclohexane system with dimensions 4.2 × 4.2 × 8.8 nm (see Figure 1a). The water/cyclohexane interface is defined as the region where the water density drops from 90 to 10%, has a width of 0.7 nm. Water molecules that overlapped with the peptide were removed and the system was energy-minimized. The biphasic cell contains 434 cyclohexane molecules and ~2000 molecules of water. In addition the Asp peptide system contains 2 Na^+ ^atoms and the peptides with -1 net charge contain 1 Na^+^.

#### Peptides in a solvated DOPC lipid bilayer

Two peptides were embedded in a pre-equilibrated (25 ns) lipid bilayer consisting of 64 molecules of DOPC (32 per leaflet). This DOPC structure is available from . Holes were generated in both sides of the lipid interface, at a location approximately suggested by the experimental data, by applying a radial force in a short MD simulation (500 ps) [20]. The two peptides were then inserted into the resulting free space. This biases the simulations towards having the peptides at the interface, which saves substantial computational efforts. Starting outside the lipid/water interface, it typically takes of the order of 20–40 ns for the peptides to bind at the interface (although some peptides are faster, some are much slower, at random), which is computationally challenging. The systems were re-solvated with 43 water molecules per lipid and then energy-minimized. The dimensions of the systems are x, y ~ 4.7 nm and z ~ 7.7 nm. The total number of atoms is ~12,000. The two peptides have different orientations in the interface, thus providing an internal control for convergence of the bilayer simulations. We will refer to the two peptides as "upper" and "lower", on the basis of their z-coordinates in the simulation, but there is no fundamental difference between the two and both should give the same results if our sampling would be complete.

### Simulation details

The MD simulations were carried out using GROMACS set of programs [21]. We used the GROMOS96 43a2 force field for the peptides and the cyclohexane [22,23] and the DOPC lipids parameter were taken from the OPLS-based force field of Berger et al. [24] combined with GROMOS87 bonded parameters and parameters for the CH1 atoms in the double bond. All carbon atoms with non-polar hydrogens are treated as united atoms, so that a cyclohexane molecule has 6 atoms and DOPC has 54 atoms. The water model used was the Simple Point Charge (SPC) [25]. Bond lengths were constrained using the LINCS algorithm [26]. Lennard-Jones interactions were calculated with a 0.9/1.4 nm twin-range cutoff. The electrostatic interactions were calculated using Particle Mesh Ewald algorithm with a cutoff of 0.9 [27]. The neighbour list was updated every 10 steps. Each component of the systems was coupled separately to a temperature bath at 300 K, using a Berendsen thermostat, with a coupling constant τ_T _= 0.1 ps [28]. The pressure was kept at 1.0 bar using pressure coupling with τ_P _= 1.0 ps [28]. In the water/cyclohexane simulation, the x and y dimensions (the area of the interface) of the system were held fixed, while the z dimension was coupled to a pressure of 1.0 bar. Two ensembles for the lipid simulations were used, NpAT and NpT. The NpT simulations were performed with anisotropic pressure coupling to 1 bar independently in x, y, z, which allows the area per lipid to fluctuate. Although this is not ideal for long simulations because the box may become elongated in either the x or y direction, in practice this did not result in problems in the 50 ns simulations in this study. For longer simulations, a scheme that scales x and y uniformly but independently from z is more desirable. Strictly speaking our method does not give an NpT ensemble. This has been discussed in detail in the literature [29,30]. In the NpAT simulations the area was fixed, while the z dimension was coupled to a pressure of 1.0 bar. Simulations were run with a 2-fs time step for the water/cyclohexane cell. The time step was 5 fs for the lipid bilayer systems using a special treatment of the hydrogens in the peptide and the aromatic rings [31]. The data was collected every picosecond. The total simulations time was 10 ns for the water/cyclohexane simulations and 50 ns for the lipid bilayers. All analyses were done with GROMACS programs. Molecular graphics were made using VMD [32].

## Authors' contributions

MPA carried out the simulations and most analyses. MPA and DPT designed the study and wrote the paper.

## Supplementary Material

Additional File 1 Trajectory of the Ile-peptide in the water-cyclohexane system (10 ns). The cyclohexane is orange, water red and white, the peptide backbone is colored pink, and the side chain atoms are colored as follows: nitrogen blue, oxygen red, carbon cyan and hydrogen white. Click here for file

Additional File 2 Trajectory of the Phe-peptide in the DOPC bilayer (50 ns) with a constant area. Water is represented by small red spheres, the lipid headgroups are shown in yellow, the tails in gray, and the side chain atoms are colored as follows: nitrogen blue, oxygen red, carbon is cyan and hydrogen is white. Click here for file
